# Is comfort food really good for the soul? A replication of Troisi and Gabriel's (2011) Study 2

**DOI:** 10.3389/fpsyg.2015.00314

**Published:** 2015-04-01

**Authors:** Lay See Ong, Hans IJzerman, Angela K.-Y. Leung

**Affiliations:** ^1^School of Social Sciences, Singapore Management University, SingaporeSingapore; ^2^School of Social and Behavioral Sciences, Tilburg UniversityTilburg, Netherlands

**Keywords:** replication, comfort food, loneliness, embodied cognition

## Abstract

We report the results of three high-powered replications of Troisi and Gabriel's (2011) idea that writing about comfort food reduces feelings of loneliness amongst securely attached individuals after a belongingness threat. We conducted our studies amongst a large group of participants (Total *N* = 649) amongst American (MTurk), Dutch (Tilburg University; TiU), and Singaporean (Singapore Management University; SMU) samples. Participants first completed an attachment style scale, followed by writing two essays for manipulating a sense of belongingness and salience of comfort food, and then reporting their loneliness levels. We did not confirm the overall effect over all three countries. However, exploratory results provide the preliminary suggestion that (1) the comfort food explanation likely holds amongst the American samples (including Troisi and Gabriel's), but not amongst the TiU and SMU samples, and potentially that (2) the TiU and SMU participants self-regulate through warmer (vs. colder) temperature foods. Both of these should be regarded with great caution as these analyses were exploratory, and because the *N*s for the different temperature foods were small. We suspect we have uncovered first cross-cultural differences in self-regulation through food, but further confirmatory work is required to understand the cultural significance of comfort food for self-regulation.

## Introduction

Troisi and Gabriel (2011) reported two studies that investigated whether associations with social relationships is what imbues comfort foods with the ability to comfort individuals. Bridging existing research on the consequences of experiencing loneliness (e.g., Baumeister and Leary, 1995; Williams, 2007) and the benefit of social surrogacy through contacts of non-human social targets (e.g., Derrick et al., 2009), Troisi and Gabriel (2011) proposed that comfort food could serve as a social surrogate. In their Study 1, participants were asked to consume chicken noodle soup^1^ or not. Supporting their prediction, participants who consumed chicken noodle soup were more likely to recall relationship-related words, as compared to those who did not consume the comfort food.

Their Study 2 involved participants answering questions about their attachment style, completing two essays, and then reporting their loneliness levels. Two key experimental manipulations were given in the essay-writing tasks. In the first essay, participants either wrote about a fight with a close other (belongingness threat condition) or listed items in their room or home (no threat condition). In a second essay, participants either wrote about their experience eating a comfort food (comfort food condition) or a new food (control food condition). Troisi and Gabriel (2011) observed that after recalling the fight episode securely attached participants reported lower loneliness levels (*M* = 1.47, *SD* = 0.45) if they were asked to think and write about their experience of eating a comfort food, as compared to those who wrote about a new food (*M* = 1.94, *SD* = 0.59), as well as those insecurely attached participants who wrote about a comfort food (*M* = 2.49, *SD* = 0.60) or a new food (*M* = 2.18, *SD* = 0.75). These findings suggested that consuming comfort food or thinking about the experience of consuming comfort food could buffer us from negative emotional consequences of social rejection. And, suggestively, how some food items become comfort foods is due to the repeated associations between the food items and the comfort presence of relational partners (e.g., a caregiver).

In the current research, we sought to replicate the findings of Study 2 from Troisi and Gabriel (2011). We chose to replicate the original Study 2 for two reasons. First, doing so will negate any potential cross-cultural problems concerning whether a specific type of food (in this case, chicken noodle soup) represents a comfort food to the participants. Because of the diverse nature of our samples (Singaporean and Dutch students in our registered replications, American MTurkers in a study conducted prior to the registration), we were unsure whether non-American participants would recognize chicken noodle soup as a comfort food. Our own pilot studies^2^ revealed that overall, a spontaneously reported comfort food for Dutch students is chocolate^3^ and the comfort food for Singaporean students is noodles. Only 37 out of 146 (25%) Singaporean students spontaneously indicated chicken noodle soup as a comfort food.

Second, whereas Study 1 revealed that consuming comfort food caused participants to recall relationship constructs, Study 2 revealed that idiosyncratically recalled comfort food lowered feelings of loneliness after securely attached participants recalled a relationship threat. As Study 2 was a conceptual extension of Study 1 by building upon the interesting link between comfort food and the internal working models of relationships, we believe that useful insights could be provided on comfort food as a social surrogate that carries important benefits both for theories on emotion regulation, as well as potential interventions, if we can confirm these findings through replications.

## Method

### Power analysis and sampling plan

Based on the effect size from the original study (Cohen's *f* = 0.27^4^), with a power of 0.95 and an alpha of 0.05, we estimated the required sample size to be 186 participants (G*Power; Faul et al., 2009). As the measurement of attachment style is an individual difference variable and we could not be certain of its prevalence in our populations, we rounded up the target sample size to 200 to increase our chances of getting a sufficient number of participants with secure attachment style.

Three separate replications were conducted in three locations: The U.S. through the Amazon Mechanical Turk (MTurk) platform, Singapore by recruiting student participants from the Singapore Management University (SMU), and the Netherlands by recruiting student participants from Tilburg University (TiU). Except for the MTurk replication that had a target sample size of 281 participants^5^, SMU and TiU were conducted with a target sample size of 200 participants each. The samples at SMU and TiU were collected after registration with this special journal issue; the sample through MTurk was collected before registration.

### Participants

Demographic information for all participants and cell sizes can be found in Table 1.

**Table 1:** **Demographic information for three replication attempts**.

**Location**	**MTurk**	**SMU**	**TiU**
*N* (Total)	426	200	181
*N* (Analyzed)	366	198	176
Ethnicity (%):			
Asian	7.4	90.4	
Hispanic	4.6	0.5	
White	74.6	3	
Others	3.8	6.1	
African American	8.5		
Native American	1.1		
Native Dutch			94.3
Afghan Dutch			0.6
Antillean Dutch			0.6
Half surinam, half Dutch			0.6
Indonesian Dutch			0.6
Iraqi			0.6
Soviet Dutch			0.6
Age (*M, (SD))*	34.54 (12.18)	21.43 (2.15)	22.59 (7.80)
Gender (% Female)	47.8	65.7	63.6
% Native English Speaker *(Dutch for TiU)*	97.3	78.8^a^	97.7
*N* (Belongingness Threat)	177	100	86
*N* (Belongingness Control)	189	98	90
*N* (Comfort Food)	244	99	90
*N* (Food Control)	122	99	86
*N* (Secure Attachment)	140	69	83
*N* (Insecure Attachment)	226	129	93

a *93.4% of participants are English speakers for 10 or more years*.

#### Amazon mechanical Turk (MTurk)

Four hundred twenty six participants were collected via the MTurk platform, instead of 281 participants, due to an oversight where only the comfort food condition was ran (but the attachment style measurement and the belongingness threat manipulation were administered as planned). As a result, we recruited additional participants in the new food condition to make up for the loss. 57 incomplete responses were discarded as (1) we were not sure if these participants underwent the manipulations successfully (i.e., they left the essay sections empty; *N* = 42), and/or (2) participants failed to provide us data for our dependent variable (i.e., they left the loneliness scale empty; *N* = 53), or (3) participants' IP addresses appeared twice (*N* = 4)^6^. Additionally, three participants were excluded from analysis as they either had no comfort food or refused to write about it. This resulted in 366 usable participants. Participants were paid USD$3.00 for their time. This study was run prior to the registration of this paper at Frontiers.

#### Singapore management university (SMU)

Two hundred participants were recruited via the university subject pool. Two participants were excluded, as one reported having no comfort food whereas the other had previously done a similar study. This resulted in 198 usable participants. Participants were paid $5 SGD (~$3.98 USD) for their participation.

#### Tilburg university (TiU)

This study was conducted as part of a class project. Although the target was to recruit 200 participants in the Psychology building who would complete the study in an isolated room, only 181 participants were recruited^7^. Five participants were excluded as they guessed the purpose of the study. This resulted in 176 usable participants. All participants participated in this study voluntarily without compensation.

### Materials and procedure

We followed the procedures of the original Study 2 as closely as possible and used the original materials provided by the authors. One minor difference was our use of the Qualtrics online survey platform instead of the original pen and paper method to administer the study. The materials can be found in Appendix A: Attachment Style scale, essay questions for belongingness threat manipulation and comfort food manipulation, State Loneliness scale, PANAS, questions about food association, and some general information to test for suspicion^8^. All reliabilities can be found in Table 2.

**Table 2:** **Reliabilities (α) of scale items for three replication attempts**.

**Scales**	**MTurk**	**SMU**	**TiU**
State belongingness^*^	0.96	0.93	0.90
State loneliness	0.97	0.94	0.90
State self-esteem 1^*^	0.91	0.82	0.87
State self-esteem 2^*^	0.95	0.94	0.88
PANAS (positive)	0.92	0.92	0.91
PANAS (negative)	0.94	0.92	0.87
Food association (comfort)	0.87	0.85	0.87
Food association (new)	0.90	0.91	0.86

Following the original study, participants first answered the Attachment Style scale. In accordance with the original study, the final question of the Attachment Style scale was used to classify participants' attachment style (see also Appendix A). Next, participants were either tasked to write an essay on a fight with a close other (belongingness threat condition) or list down items in their residence (no threat condition) for 6 min as per original design. A timer was ran in the background of the task to remind participants of the time. Once the 6 min were up, the survey automatically proceeded to the next task, where the participants were either asked to write about the experience of eating a comfort food, or trying a new food, before completing the rest of the scales.

#### Coding of food essays

Following Troisi and Gabriel's coding instructions, undergraduate research assistants—blind to the purpose and hypothesis of the study—coded the food essays. For each replication attempt, different pairs of research assistants coded the essays (and in the case of the MTurk and TiU versions, the coding was done by groups of students). Upon completion of the initial round of coding, the first (SMU) and second (MTurk, TiU) authors highlighted the discrepancies between the coders and had the coders meet to resolve their inconsistencies until they reached an agreement. For items that were based on the subjective ratings of the coders, we averaged the ratings between the two coders.

#### Location

Due to the nature of the MTurk online platform, we are unable to comment on the nature of the environment in which participants had completed the study. At SMU, all study sessions were conducted in individual cubicles inside the psychology lab, where participants completed the study alone. In TiU, the aim was to have participants complete the study individually. However, as it was difficult to recruit enough participants for the lab study, halfway through data collection, we allowed participants to complete the study at home. For these participants, we explicitly urged them to complete the study by themselves in a quiet location. In addition, at the end of the survey we asked them to confirm whether or not they completed the study in one setting and without distraction^9^. We will specify the location of data collection in the respective result sections.

## Results

In the following results section, we first report our findings from the proposed analysis plan, followed by exploratory analyses to potentially explain differences between the original and current reports (cf. Wagenmakers et al., 2012; Brandt et al., 2014).

### Confirmatory comparisons between comfort and new food essays

#### MTurk

Following the original study, an analysis on the coded essays indicated that new foods (*M* = 0.04, *SD* = 0.08) were less likely to be identified as a favorite food, a family tradition, a cultural tradition, something eaten during a holiday, something eaten during a significant family occasion, part of a participant's past, or a reminder of home, as compared to comfort foods (*M* = 0.08, *SD* = 0.12), *t*_(364)_ = 3.35, *p* = 0.001, *d* = 0.38^10^, 95% C.I. of *d* [0.15, 0.59].

#### SMU

Not confirming the original study, the coded essays did not indicate that new foods (*M* = 0.03, *SD* = 0.06) were less likely to be identified as a favorite food, a family tradition, a cultural tradition, something eaten during a holiday, something eaten during a significant family occasion, part of a participant's past, or a reminder of home, as compared to comfort foods (*M* = 0.02, *SD* = 0.05), *t*_(196)_ = −0.98, *p* = 0.33, *d* = −0.14, 95% C.I. of *d* [−0.42, 0.14].

#### TiU

The coded essays indicated that new foods (*M* = 0.04, *SD* = 0.07) were only marginally significantly less likely to be identified as a favorite food, a family tradition, a cultural tradition, something eaten during a holiday, something eaten during a significant family occasion, part of a participant's past, or a reminder of home, as compared to comfort foods (*M* = 0.07, *SD* = 0.11), *t*_(174)_ = 1.70, *p* = 0.09, *d* = 0.26, 95% C.I. of *d* [−0.04, 0.55], though the pattern was in line with the original results and the MTurk sample.

### Original authors' analysis plan

We conducted the analysis plan described by the original authors. We report all ANOVA and *t*-test analyses in Appendix B^11^. Means from each cell sizes can be found in Appendix E. The analyses from our replication studies did not produce the same findings as the original authors. Specifically, the hypothesized 3-way interaction was not found in the MTurk sample [Cohen's *f*^2^ = 0.05, *F*_(1,358)_ = 1.08, *p* = 0.30, η^2^_*p*_ = 0.003], the SMU sample [Cohen's *f*^2^ = 0.11, *F*_(1, 190)_ = 2.54, *p* = 0.11, η^2^_*p*_ = 0.013], nor the TiU sample [Cohen's *f*^2^ = 0.006, *F*_(1, 167)_ = 0.007, *p* = 0.93, η^2^_*p*_ = 0.00004]. The results were comparable when mood was controlled for: MTurk [Cohen's *f*^2^ = 0.04, *F*_(1, 356)_ = 0.57, *p* = 0.45, η^2^_*p*_ = 0.002], SMU [Cohen's *f*^2^ = 0.04, *F*_(1, 188)_ = 0.35, *p* = 0.55, η^2^_*p*_ = 0.002], TiU [Cohen's *f*^2^ = 0.04, *F*_(1, 165)_ = 0.38, *p* = 0.54, η^2^_*p*_ = 0.002].

### Planned comparisons

Despite the lack of the critical 3-way interaction, we conducted planned contrast comparisons in order to provide the confirmatory analyses. For each sample, we conducted four planned comparisons to investigate whether the original findings could be detected^12^. These are in accordance to our proposed analysis plan, except for Comparison 4, which was decided after our preregistration document. To enable us to compare the range of *d*s of the supported contrasts with the original study, we also calculated the CIs of the *d*s of the original data (see below for further explanations).

Planned comparisons of replication attempts:

For each set of contrast analysis, we follow this coding for conditions:

1 = Secure, New Food, Belong Control
2 = Insecure, New Food, Belong control
3 = Secure, Comfort Food, Belong Control
4 = Insecure, Comfort Food, Belong Control
5 = Secure, New Food, Belong Threat
6 = Insecure, New Food, Belong Threat
7 = Secure, Comfort Food, Belong Threat
8 = Insecure, Comfort Food, Belong Threat

MTurk Replication:

The contrasts of interest are:

**Table:** 

	**Expectation**	**Group A**	**Group B**	***t-value***	***d*^13^**	**CI (95%) of d**
Comparison 1 (5 vs. 7) (*p* = 0.12)	Threatened, securely attached participants should experience lower levels of loneliness if they wrote about comfort food as compared to if they wrote about new food.	5 (*M* = 2.14, *SD* = 0.77)	7 (*M* = 1.81, *SD* = 0.72)	*t*_(358)_ = −1.58	−0.38	[−0.86 to 0.10] Original study [−1.50 to 0.05]
Comparison 2 (7 vs. 8) (*p* = 0.001)	After writing about comfort food, threatened, securely attached participants should experience lower levels of loneliness as compared to insecurely attached.	7 (*M* = 1.81, *SD* = 0.72^14^)	8 (*M* = 2.83, *SD* = 0.91^15^)	*t*_(358)_ = −6.47	−1.20	[−1.59 to −0.80] Original study [−2.40 to −0.77]
Comparison 3 (3 vs. 7) (*p* = 0.16)	After writing about comfort food, securely attached participants who underwent a belongingness threat should not differ in loneliness level from their counterparts in the no threat condition (null hypothesis).	3 (*M* = 1.57, *SD* = 0.58)	7 (*M* = 1.81, *SD* = 0.72)	*t*_(358)_ = −1.40	−0.28	[−0.68 to 0.12] Original study [−0.44 to 1.08]
Comparison 4 ^*^new^*^ (1 vs. 5) (*p* = 0.12)	Those asked to write about a belongingness threat should report greater loneliness levels, as compared to those who wrote about things in their apartment.	1 (*M* = 1.70, *SD* = 0.67)	5 (*M* = 2.14, *SD* = 0.77)	*t*_(358)_ = 1.58	0.52	[−0.13 to 1.17] Original study [−0.16 to 1.63]

Singapore Replication:

The contrasts of interest are:

**Table:** 

	**Expectation**	**Group A**	**Group B**	***t-value***	***d***	**CI (95%) of d**
Comparison 1 (5 vs. 7) (*p* = 0.33)	Threatened, securely attached participants should experience lower levels of loneliness if they wrote about comfort food as compared to if they wrote about new food.	5 (*M* = 1.88, *SD* = 0.43)	7 (*M* = 2.11, *SD* = 0.57)	*t*_(190)_ = 0.97	0.32	[−0.33 to 0.97] Original study [−1.50 to 0.05]
Comparison 2 (7 vs. 8) (*p* = 0.32)	After writing about comfort food, threatened, securely attached participants should experience lower levels of loneliness as compared to insecurely attached.	7 (*M* = 2.11, *SD* = 0.57)	8 (*M* = 2.32, *SD* = 0.68)	*t*_(190)_ = −1.0	−0.29	[−0.86 to 0.28] Original study [−2.40 to −0.77]
Comparison 3 (3 vs. 7) (*p* = 0.73)	After writing about comfort food, securely attached participants who underwent a belongingness threat should not differ in loneliness level from their counterparts in the no threat condition (null hypothesis).	3 (*M* = 2.02, *SD* = 0.49)	7 (*M* = 2.11, *SD* = 0.57)	*t*_(190)_ = −0.35	−0.12	[−0.80 to 0.56] Original study [−0.44 to 1.08]
Comparison 4 ^*^new^*^ (1 vs. 5) (*p* = 0.85)	Those asked to write about a belongingness threat should report greater loneliness levels, as compared to those who wrote about things in their apartment.	1 (*M* = 1.92, *SD* = 0.59)	5 (*M* = 1.88, *SD* = 0.43)	*t*_(190)_ = −0.19	−0.06	[−0.73 to 0.60] Original study [−0.16 to 1.63]

Netherlands Replication:

The contrasts of interest are:

**Table:** 

	**Expectation**	**Group A**	**Group B**	***t-value***	***d***	**CI (95%) of d**
Comparison 1 (5 vs. 7) (*p* =0.98)	Threatened, securely attached participants should experience lower levels of loneliness if they wrote about comfort food as compared to if they wrote about new food.	5 (*M* = 1.65, *SD* = 0.28)	7 (*M* = 1.65, *SD* = 0.36)	*t*_(167)_ = 0.02	0.007	[−0.27 to 0.27] Original study [−1.50 to 0.05]
Comparison 2 (7 vs. 8) (*p* = 0.001)	After writing about comfort food, threatened, securely attached participants should experience lower levels of loneliness as compared to insecurely attached.	7 (*M* = 1.65, *SD* = 0.36)	8 (*M* = 2.18, *SD* = 0.69)	*t*_(167)_ = −3.32	−1.03	[−1.67 to −0.38] Original study [−2.40 to −0.77]
Comparison 3 (3 vs. 7) (*p* = 0.54)	After writing about comfort food, securely attached participants who underwent a belongingness threat should not differ in loneliness level from their counterparts in the no threat condition (null hypothesis).	3 (*M* = 1.74, *SD* = 0.36)	7 (*M* = 1.65, *SD* = 0.36)	*t*_(167)_ = 0.61	0.18	[−0.40 to 0.76] Original study [−0.44 to 1.08]
Comparison 4 ^*^new^*^ (1 vs. 5) (*p* = 0.73)	Those asked to experience belongingness threat should report greater loneliness levels, as compared to those who did not experience the threat.	1 (*M* = 1.71, *SD* = 0.29)	5 (*M* = 1.65, *SD* = 0.28)	*t*_(167)_ = −0.34	−0.11	[−0.77 to 0.54] Original study [−0.16 to 1.63]

**Comparison 1:**
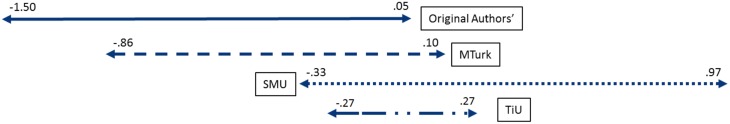
**Threatened, securely attached participants should experience lower levels of loneliness if they wrote about comfort food as compared to if they wrote about new food**.

**Comparison 2:**
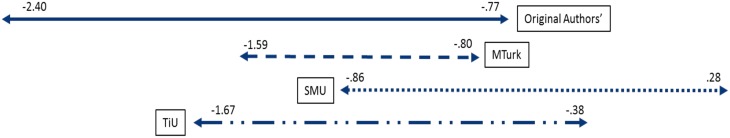
**After writing about comfort food, threatened, securely attached participants should experience lower levels of loneliness as compared to insecurely attached**.

**Comparison 3:**
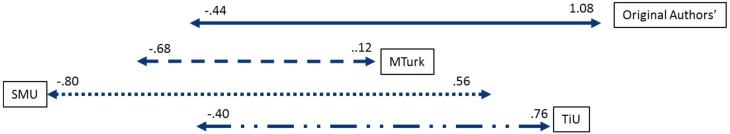
**After writing about comfort food, securely attached participants who underwent a belongingness threat should not differ in loneliness level from their counterparts in the no threat condition (null hypothesis)**.

**Comparison 4:**
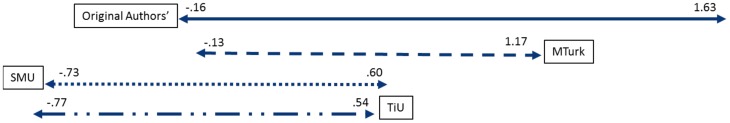
**Those asked to write about a belongingness threat should report greater loneliness levels, as compared to those who wrote about things in their apartment**.

Although the manipulations seemed to work for the MTurk sample, it is important to note that in this sample we did not ask whether others were present, and this environment generally introduces a greater chance for noise (like the participant eating). Our interpretation is that this is a more conservative test of Troisi and Gabriel's (2011) hypothesis, as our lack of being able to control for such factors likely introduced more noise^16^.

### Simonsohn's replication evaluation plan

Following our proposal, we conducted the replication evaluation plan as described in Simonsohn (in press) for our 3-way interactions (Appendix E). Utilizing the analysis on the simple effect of the original finding (i.e., social threat makes people feel lonelier), the MTurk and TiU data indicate that the null of a detectable (d33%) effect was rejected, implying that the effect that is being investigated is smaller than small (i.e., non-existent). The SMU data did not reject the null (*p* = 0.06), indicating the inability for this data to inform us about the effect size of this finding.

In addition to Simonsohn's evaluation method, we also examined the differences between the overall contrasts of the original study and the replication attempts. Simonsohn's evaluation method did not permit us to do comparable calculations for contrast comparisons, so we compared the overlap between the CI of each contrast. Based on Simonsohn's (in press) work, we realize that this may not be ideal, but given that Troisi and Gabriel's (2011) sample size was substantial, it at least provides us some information regarding the nature of the replication. Summarized in the diagrams below, we observed that for the planned contrasts, all CIs of the MTurk replication attempts overlapped with the original study. Notably, two of the original contrasts were likely only marginally significant, while the most crucial comparison, Comparison 2 (which reflects differences between securely attached vs. insecurely attached participants after a belongingness threat and writing about comfort food) did not include 0 and was replicated in the MTurk sample and the TiU sample (however, the TiU sample sometimes appeared more like the SMU sample, and sometimes like the MTurk sample, see e.g., Comparison 4).

### Exploratory analyses

From both our manipulation check (in which only the MTurk sample was significant as to whether the food was considered a veritable comfort food) and our confirmatory analyses, it appeared that we have detected cultural differences in the meaning of comfort food. The nature of its status is less clear in our TiU sample; the manipulation check was marginally significant, and the data from the confirmatory analyses left us with a mixed picture. Our own inference is that our Dutch sample does not have as strong an (semantic) association with the idea of comfort food as our American (and Troisi and Gabriel's American) sample has.

To explore whether our cross-cultural intuitions may have some grounding, we conducted several exploratory analyses. We intuited that our Dutch and Singapore samples were comparable, and that Troisi and Gabriel's and our MTurk samples were comparable. We first ran an analysis comparing our TiU and SMU samples (coded as −1) with our MTurk sample (coded as 2) on the 3-way interaction between belongingness threat, food condition, and attachment style. This 4-way interaction gave us a non-significant result [*F*_(1, 633)_ = 2.28, *p* = 0.131, η^2^_*p*_ = 0.004. This may partly be due to low power to detect such a 4-way effect, and the relative ambivalent nature of the TiU sample on comfort foods [only comparing the SMU and MTurk gave a comparable, but somewhat stronger effect, *F*_(1, 548)_ = 3.21, *p* = 0.074, η^2^_*p*_ = 0.006]. If we ran the 4-way interaction with Troisi and Gabriel's participants included, our results were comparable [*F*_(1, 730)_ = 3.36, *p* = 0.067, η^2^_*p*_ = 0.005]. Alongside the confirmatory analyses, we thus tentatively conclude that the comfort food effects “work” for the two American samples, but not the SMU and TiU samples.

In order to investigate an alternative in self-regulation across cultures after a belongingness threat, we compared the temperature of the food (which was coded across these samples as well). Social thermoregulation has been thought to be one way via which people can downregulate negative emotional states (particularly if they are rooted in belongingness concerns; IJzerman and Semin, 2009; IJzerman and Koole, 2011; Beckes et al., 2014). When we ran the 4-way interaction, comparing both American samples to TiU and SMU samples, secure versus insecure attachment, belongingness threat vs. control, and cold dishes versus room temperature and hot dishes, we found a marginal 4-way interaction, *F*_(1, 725)_ = 3.14, *p* = 0.077, η^2^_*p*_ = 0.004, with SMU/TiU participants scoring lower on loneliness when recalling higher temperature foods (*M* = 1.93, *SD* = 0.49) than lower temperature foods (*M* = 2.24, *SD* = 0.59) when they are secure and in a belongingness threat. These differences were not there among American participants (colder: *M* = 1.76, *SD* = 0.84; warmer: *M* = 1.89, *SD* = 0.68). Although we would like to suggest that TiU and SMU participants—seemingly not aware of the “comfort food” association—rely on less verbalized mechanisms (i.e., temperature of the food) to self-regulate themselves, samples were unfortunately small (for TiU and SMU samples—warm, secure, belongingness threat: *N* = 29; cold, secure, belongingness threat: *N* = 8). In other words, these first explorations need confirmations.

## Discussion

Food forms an integral part of our lives. Prior research indicated a close relationship between food intake and physiological effects (e.g., Markus et al., 1998; Oliver et al., 2000). In particular, food has also been closely connected to self regulation. In a daily diary study by Macht and Dettmer (2006), participants reported greater joy and elevated moods after the consumption of chocolate. In yet another study, Markus et al. (1998) showed that a high carbohydrate and low protein diet resulted in lowered feelings of helplessness and depression as it raised the level of serotonin. Troisi and Gabriel (2011) extended these findings by integrating this area of research with an embodied cognition framework, thereby shedding new light on the self regulatory implication potentially afforded by comfort food. We attempted three high-powered replications on Troisi and Gabriel (2011) Study 2. Our data showed no overall effect of comfort food on reducing loneliness levels after having participants recall a social rejection event. Specifically, no difference emerged between the securely attached and insecurely attached participants. However, if we separated our samples by country, we did find a comparable pattern in our MTurk samples, but not in our TiU and SMU samples (although the Dutch sample was less clear).

Our exploratory analyses suggested potential cross cultural differences of the comfort food manipulation. Interestingly enough, when we casually inquired amongst our Dutch colleagues, none seemed to be aware of comparable Dutch words for comfort food (or outdated ones, like “a bakkie troost”). In the Dutch questionnaire, we used the English term in the Dutch questionnaire, which may be the reason for the Dutch effects to turn up somewhat ambivalent. However, our exploratory results were weak, and thus require further investigation to know whether our impressions of potential cross-cultural differences hold up. Finally, we suspect temperature of the food plays some role in self-regulation from negative states, but—beyond the analyses being exploratory—our samples were too small to draw any firm conclusions.

Of course, it is also possible that the effect found by the original authors does not exist and that caused the weak replication results. However, our null finding could also be due to certain specificities associated with our replication set-up. For example, our participants' demographics (i.e., older Americans in the MTurk sample, Singaporean undergraduates in the SMU sample, and Dutch undergraduates in the TiU sample) differed from those in the original study (i.e., American undergraduates). Our MTurk participants also were in a different study environment than the participants in the original study, which we think was the biggest reason for it being a somewhat smaller effect than the original study. As all of these reasons are speculative at best, and we encourage future research to tease apart these possible reasons to investigate the actual effect and whether the methodology is applicable in non-US samples.

All in all, we think that our large-scale replication effort uncovered some first important differences in how people regulate their emotional states through food, such that food across cultures is imbued with a different purpose for self-regulation. We think that this replication effort thus did not only provide a service in providing a more accurate estimate of the effect size, but also provides a first step in building a more comprehensive theory of the role of food in people's lives.

### Conflict of interest statement

The authors declare that the research was conducted in the absence of any commercial or financial relationships that could be construed as a potential conflict of interest.

## Footnotes

^1^Four to six weeks prior to this study, participants were surveyed to ensure that chicken noodle soup was regarded as a comfort food.

^2^As we originally wanted to replicate Study 1, these studies were conducted because we were not convinced that chicken noodle soup would be considered a comfort food outside of the U.S. However, we chose to switch to replicating Study 2 mainly because it removes this problem of cultural differences in comfort food.

^3^It is also interesting to note that in a Dutch study investigating emotional eating (Evers et al., 2010), they used chocolate, crisps, and cookies as the comfort food in two studies.

^4^We calculated Cohen's *f*^2^ using G^*^Power. This (and subsequent) Cohen's *f*^2^ was not from the original authors. We calculated this based on their reported η^2^.

^5^The MTurk replication was conducted before our proposal was submitted to the journal editors. During sample size estimation, we made a mistake in G^*^Power by using a fixed effects model to calculate the a prior power, rather than one based on interactions.

^6^We are aware that double IP addresses may indicate something else than taking the study twice (e.g., participating in the same computer lab). In order to test our hypothesis as conservatively as possible, and to avoid any foreknowledge of the study, we excluded these participants from our sample.

^7^Data collection during this part of the year is difficult, as many other groups of students were collecting their data at that time.

^8^In addition, there were other scales included in the original study that were not reported in the article (we indicated these scales in Appendix A by providing an ^*^ besides the title): State Belongingness scale, State Self-esteem scales 1 and 2. According to Troisi, the two self-esteem scales were added as manipulation checks to ensure that the manipulation of activating comfort food would only affect the levels of loneliness, but not self-esteem (and their results were as expected). The State Belongingness scale was added, because it was conceptually similar to loneliness in a reversed direction and the activation of comfort food was expected to have an effect on state belongingness (but their results did not show this expected pattern). The authors did not report these scales in their original paper due to word-count considerations. In this replication attempt, we have included these unmentioned scales into the study so as to follow the original procedures strictly. All reliabilities can be found in Table 2.

^9^Rerunning the analysis by excluding those who did not complete the survey at the university (*N* = 93), or who were not alone when completing the survey (*N* = 14) did not lead to different results.

^10^We calculated Cohen's *d* using this online calculator: http://www.campbellcollaboration.org/escalc/html/EffectSizeCalculator-SMD2.php.

^11^Analyses for the TiU sample with the different exclusions are reported in Appendix C.

^12^Contrast comparisons for the TiU sample excluding those who did not complete the study in the university lab or did not do the study alone are reported in Appendix D.

^13^All Cohen's *d* s and C.I.s of the contrast comparisons were calculated using Karl Wuensch's SPSS syntax (http://core.ecu.edu/psyc/wuenschk/SPSS/SPSS-Programs.htm).

^14^In the previous proposal, this was reported wrongly as *SD* = 0.91.

^15^In the previous proposal, this was reported wrongly as *SD* = 0.95.

^16^According to Gabriel who previously reviewed our proposal, the control over the setting of the study is extremely important to ensure no other variables influences the dependent variable.
