# Supplementary material for: Is comfort food really good for the soul? A replication of Troisi and Gabriel's (2011) Study 2
Source: Front Psychol. 2015 Apr 1;6:314. doi: 10.3389/fpsyg.2015.00314 (PMC4381504; doi:10.3389/fpsyg.2015.00314)
Supplement: Supplementary file 1 [file DataSheet1.PDF]

Appendix A
Original Study Materials from Jordan Troisi

Attachment Scale

INSTRUCTIONS: Please read the following statements about your general relationship style. Indicate how characteristic each of the following statements is of you according to the following scale:

1	Not at all like me
2	
3	
4	
5	Somewhat like me
6	
7	
8	
9	Very much like me

1. It is easy for me to be emotionally close to others. I am comfortable depending on others and having others depend on me. I don't worry about being alone or having others not accept me. (attach1)

2. I am comfortable without close emotional relationships. It is very important to me to feel independent and self-sufficient, and I prefer not to depend on others or have others depend on me. (attach2)

3. I want to be completely emotionally intimate with others, but I often find that others are reluctant to get as close as I would like. I am uncomfortable being without close relationships, but I sometimes worry that others don't value me as much as I value them. (attach3)

4. I am uncomfortable getting close to others. I want emotionally close relationships, but I find it difficult to trust others completely, or to depend on them. I worry that I will be hurt if I allow myself to become too close to others. (attach4)

5. Next, please indicate which of the above relationship styles (paragraphs 1-4) is most characteristic of you.

NOTE: the variable “secure” was created by a yes/no dichotomy of whether participants indicated the secure paragraph was most characteristic of them (“yes”) or if some other attachment paragraph was most characteristic of them (“no”).

Belonging Threat Essay Condition

Please think of a time when you had a fight with someone close to you. Imagine yourself in the situation, and try to relive it. Describe what happened in as much detail as possible, and describe how you felt at the time. You will have six minutes to write about this event.

(AFTER COMPLETING THE ESSAY: Participants reported the nature of the relationship with the person described. This variable is labeled "reject2", with response options as below:
1 = good friend
2 = best friend
3 = family member
4 = current romantic partner
5 = past romantic partner
6 = other)

Control Essay Condition

Please list as many items as you can remember in your bedroom, dorm room, or apartment. You will have six minutes.

Comfort Food Essay

Please think of a time when you ate some of what you consider your comfort foods. Describe the food in as much detail as possible. What food(s) did you eat? What did the food taste like? What did it smell like? Was it filling? Was it cold or warm? What were the flavors in it? How did you feel when you were eating the food? Write everything you can think of about the experience.

Control Food Essay

Please think of a time when you tried a new food that you had never eaten before. Describe the food in as much detail as possible. What food(s) did you eat? What did the food taste like? What did it smell like? Was it filling? Was it cold or warm? What were the flavors in it? How did you feel when you were eating the food? Write everything you can think of about the experience.

State Belongingness *

Please answer the following questions about how you feel right now using the scale below.

1 = strongly disagree
2
3
4 = neutral
5
6
7 = strongly agree

1. Right now, I feel like I fit in.
2. At this moment, I feel accepted by others.
3. Right now, I feel rejected.
4. I feel like nobody cares about me right now.
5. Right now, I feel connected to others.
6. At the moment, I feel like I belong.
7. I feel alone right now.
8. At this moment, I feel excluded by others.
9. Right now, I don't feel close to others.
10. Right now, I feel isolated from others.

(These are variables “sbel01”-“sbel10”)

(Reverse-scored variables are numbers 3, 4, 7, 8, 9, and 10, recoded as “sbel03new”, “sbel04new”, and so on.)

(Overall scale mean including reverse-scored variables is “Sbel”.)

State Loneliness

Please complete the following items using the scale below.

1 = not at all true of
2 = a little true
3 = somewhat true
4 = very true
5 = extremely true

1. Right now I feel that I am "in tune" with the people in my life.
2. At the moment, I feel that I lack companionship.
3. Right now, I feel like there is no one I can turn to.
4. I feel alone right now.
5. I feel like I am part of a group of friends.
6. At the moment, I feel like I have a lot in common with the people in my life.
7. I feel like I am no longer close to anyone right now.
8. I feel like my interests are not shared by the other people in my life right now.
9. Right now I feel outgoing and friendly.
10. I feel close to the people in my life at the moment.
11. I feel left out right now.
12. At the moment I feel like my relationships with others are not meaningful.
13. Right now I feel like no one really knows me well.
14. Right now I feel isolated from others.
15. I feel like I could find companionship if I wanted it right now.
16. At the moment I feel like there are people who understand me.
17. I feel shy right now.
18. Right now I feel like, even though there are people in my life, they're not really with me.
19. At the moment, I feel like there are people in my life that I can talk to.
20. Right now, I feel like there are people in my life I can turn to.

(These are variables “slone01”-“slone20”)

(Reverse-scored variables are numbers 1, 5, 6, 9, 10, 15, 16, 19, and 20, recoded as “slone01new”, “slone05new”, and so on.)

(Overall scale mean including reverse-scored variables is “Slone”.)

State Self-Esteem Scale 1 *

Please think about each emotion word that follows. How much do you feel each emotion right now?

1 = not at all
2 = a little bit
3 = somewhat
4 = very
5 = extremely

1. Right now, I feel ________ proud.
2. Right now, I feel ________ anxious.
3. Right now, I feel ________ depressed.
4. Right now, I feel ________ upset.
5. Right now, I feel ________ calm.
6. Right now, I feel ________ ashamed.
7. Right now, I feel ________ embarrassed.
8. Right now, I feel ________ relaxed.
9. Right now, I feel ________ happy.
10. Right now, I feel ________ self-confident.

(These are variables “sse01”-“sse10”)

(Reverse-scored variables are numbers 2, 3, 4, 6, and 7 , recoded as “sse02new”, “sse03new”, and so on.)

(Overall scale mean including reverse-scored variables is “SSE”.)

State Self-Esteem Scale 2 *

INSTRUCTIONS: The following are a series of statements about how you currently feel. Please indicate how you feel **right now** by indicating your extent of agreement with each item.

1 = strongly disagree
4 = neutral
7 = strongly agree

1. I feel confident about my abilities.
2. I am worried about whether I am regarded as a success or failure.
3. I feel satisfied with the way my body looks right now.
4. I feel frustrated or rattled about my performance.
5. I feel that I am having trouble understanding things that I read.
6. I feel that others respect and admire me.
7. I am dissatisfied with my weight.
8. I feel self-conscious.
9. I feel as smart as others.
10. I feel displeased with myself.
11. I feel good about myself.
12. I am pleased with my appearance right now.
13. I am worried about what other people think of me.
14. I feel confident that I understand things.
15. I feel inferior to others at this moment.
16. I feel unattractive.
17. I feel concerned about the impression I am making.
18. I feel that I have less scholastic ability right now than others.
19. I feel like I'm not doing well.
20. I am worried about looking foolish.

(These are variables "state01"-"state20")

(Reverse-scored variables are numbers 2, 4, 5, 7, 8, 10, 13, 15, 16, 17, 18, 19, and 20 recoded as "state02new", "state04new", and so on.)

(Overall scale mean including reverse-scored variables is "stateSE".)

PANAS

Directions
This scale consists of a number of words that describe different feelings and emotions. Read each item and then circle the appropriate answer next to that word. Indicate to what extent you feel this way right now.

Use the following scale to record your answers.

(1) = Very slightly or not at all (2) = A little (3) = Moderately (4) = Quite a bit (5) = Extremely

	**Very slightly or not at all**	A little	**Moderatel y**	**Quite a bit**	Extremely
1. Interested	1	2	3	4	5
2. Distressed	1	2	3	4	5
3. Excited	1	2	3	4	5
4. Upset	1	2	3	4	5
5. Strong	1	2	3	4	5
6. Guilty	1	2	3	4	5
7. Scared	1	2	3	4	5
8. Hostile	1	2	3	4	5
9. Enthusiastic	1	2	3	4	5
10. Proud	1	2	3	4	5
11. Irritable	1	2	3	4	5
12. Alert	1	2	3	4	5
13. Ashamed	1	2	3	4	5
14. Inspired	1	2	3	4	5
15. Nervous	1	2	3	4	5
16. Determined	1	2	3	4	5
17. Attentive	1	2	3	4	5

18. Jittery	1	2	3	4	5
19. Active	1	2	3	4	5
20. Afraid	1	2	3	4	5

(These are variables “mood01”-“mood20”)

(Positive affect scale computed as “posaff”; negative affect scale computed as “negaff”)

Food Associations

Please answer the following items about comfort foods using the scale provided.

1 = strongly disagree
2
3
4 = neutral
5
6
7 = strongly agree

1. Comfort foods remind me of my childhood
2. Comfort foods remind me of positive memories
3. Comfort foods remind me of negative memories
4. Comfort foods make me feel nostalgic
5. Comfort foods remind me of my family
6. Comfort foods remind me of close others
7. Comfort foods remind me of holidays or special events
8. Comfort foods remind me of home

(These are variables "food1"-"food8". A mean of items 1, 2, 4, 5, 6, 7, and 8 was computed to create the "comfood" variable)

Please answer the following items about non-comfort foods using the scale provided.

1 = strongly disagree
2
3
4 = neutral
5
6
7 = strongly agree

9. Non-comfort food reminds me of my childhood
10. Non-comfort foods remind me of positive memories
11. Non-comfort foods remind me of negative memories
12. Non-comfort foods make me feel nostalgic
13. Non-comfort foods remind me of my family

14. Non-comfort foods remind me of close others

15. Non-comfort foods remind me of holidays or special events

16. Non-comfort foods remind me of home

(These are variables “food9”-“food16”. A mean of items 9, 10, 12, 13, 14, 15, and 16 was computed to create the “nonfood” variable)

General Information

Think back to the first essay you wrote at the beginning of this study. What was it about? (mnp1)

Think back to the second essay you wrote at the beginning of this study. What was it about (mnp2)

For the second essay you wrote about at the beginning of this study, we had you write about a food experience. How long ago did this food experience occur (mnp3):
1 = within the last 3 months
2 = between 3 and 6 months ago
3 = between 6 and 12 months ago
4 = between 1 year and 2 years ago
5 = between 2 and 3 years ago
6 = between 3 and 4 years ago
7 = more than 4 years ago

For the essay about the food experience, who ate the food with you? (mnpwho)

Did you find anything odd about this study overall? (susp1) (controlled for in ANCOVA analysis)

If yes to the above, what was odd about the study? (susp2)

Had you heard about this study before coming into the lab today? (susp3)

What did you hear about the study before coming into the lab today? (susp4)

What did you think the study was about? (mnp5)

What is your gender? (sex)   Male   Female

How old are you? (age) ______

What is your ethnicity? (ethn1) American Indian___   African-American___
Asian___   Hispanic___ White___   Other___ (please specify: ethn2)
____

Is English your first language? (eng) Yes___   No___

How many years have you spoken English? (engdur) ______

The informed consent form

Recall and Social Stimuli
Informed Consent Form

Introduction: You are invited to participate in a research study titled "Recall and Social Stimuli." This study is being conducted by Associate Professor Angela Leung, and Laysee Ong from the School of Social Sciences, Singapore Management University.

Volunteer Status: Your participation in this study is completely voluntary. You may refuse to participate or withdraw from the study at any time without penalty or any effect on your present or future relationship to SMU. In addition, you may decline to answer any question that you do not want to answer. Your participation, or non-participation, in the study will not affect other relationships or services you are entitled to as a student of SMU. You have the right to refuse to answer particular questions. You have the right to withdraw your data at the end of the session, or at a later time.

Purpose of the Study: The purpose of the study is to examine relationships between recall and social stimuli. Additionally, we will be looking at aspects of the self-concept and personality.

Description of the Study: You will be asked to supply some information about yourself, including aspects of your personality and how you feel about different aspects of your self-concept. You will also be asked to recall information from the past.

Risks: Some participants may be upset by some of the questions and procedures in this study. If this occurs, please know that you may withdraw from the study at any point in time and that you may refuse to answer any question that you do not want to answer. Additionally, if you feel uncomfortable or upset at any point during the experiment, please let the experimenter know. Please also know that your responses will be locked in a storage cabinet and kept completely confidential. In any reports or presentations of this research, participants will not be identified individually.

Benefits: This experiment has the potential benefit of familiarizing you with the experimental methodology and extending recent psychological findings.

Time Commitment: Your participation in this study will take approximately 30 minutes.

Compensation: You will receive S$5 for taking part in the study.

Privacy: Recruitment and data collection procedures are designed to protect the privacy of participants. Participants have signed-up to participate in the study using a web-based program that the psychology department has in place and routinely makes us of. This program allows researchers to view the names of participants who have signed-up for the study. Participants' names will only be used to ensure that researchers have the correct participant for each study session (i.e., the participant

who signed up is the one who will participate in the study). At the end of the semester, it is routine practice that participant sign-up information is deleted from this program so that students' privacy is protected. Once data collection has begun, participants will be identified by an arbitrary number only.

<u>Confidentiality:</u> Your individual privacy will be maintained in all published and written data resulting from the study. Experimental materials will be coded with an identification number only, so that your name will not be attached to the materials. All materials will be locked in the psychology lab or faculty office. All identifying information will be removed from the experimental materials and destroyed. There will be no way to link your name with any of the questionnaires or materials that you complete during this experiment.
Any questions, concerns or complaints that you may have about this study can be answered by Laysee Ong. She can be reached by email at laysee.ong.2010@smu.edu.sg, through the School of Social Sciences, Singapore Mangement University, 90 Stamford Road, Level 4, Singapore 178903.

If you have any questions about your rights as a participant in a research project, or questions, concerns or complaints about the research and wish to speak with someone who is not a member of the research team, you should contact (anonymously, if you wish) the Institutional Review Board, Office of Research, SMU Administrative Building, 81 Victoria Street, Singapore 188065, email irb@smu.edu.sg, phone: 68281925.

<u>Before you sign this document:</u> By signing below you are agreeing to participate in a research study. Make sure that any questions have been answered to your satisfaction and that you have a thorough understanding of the study. If you decide to participate in this research study, a copy of this document will be given to you.

I agree to participate in this research.

________________________________ __________________
Participant Signature Date (mm/dd/yyyy)

I certify that I obtained the consent of the participant whose signature is above. I understand that I must give a signed copy of the informed consent form to the participant, and keep the original copy in laboratory files for 3 years after completion of the research project.

________________________________ __________________
Experimenter Signature Date (mm/dd/yyyy)

The debriefing form

DEBRIEFING FORM

Thank you for participating in this study. We appreciate the time you took to complete it. Now, we would like to tell you a little bit more about the nature of the study.

Past research has demonstrated that when people feel a lowered sense of belonging, they seek to connect with others. Some participants in this study were asked to think write about a time in which they got in a fight with a close other (others, in a control condition, were asked to list items in their residence). After this first essay, some participants were asked to write about a time in which they ate comfort food, and others were asked to write about a time when they had food that wasn't particularly special to them.

Comfort foods are typically associated with family, holidays, and special occasions. Because of this, we believe that thinking about comfort foods might get people to think about close others, and thus relieve hurt feelings of belonging. To assess this, you then completed a series of questionnaires measuring mood, self-esteem, and feelings of belonging.

Now that you have been informed of the true nature of this study, we wish to give you the opportunity to withdraw your data if you so choose. If you elect to withdraw your data, the principal investigator will delete all the data you have provided within 24 hours. Furthermore, even if you choose to withdraw your data, you will not be penalized in any way; you will still receive full credit for participating in this study.

Any questions, concerns or complaints that you may have about this study can be answered by Laysee Ong. She can be reached by email at laysee.ong.2010@smu.edu.sg, and through the School of Social Sciences, Singapore Mangement University, 90 Stamford Road, Level 4, Singapore 178903. Again, thank you for participating in our study.

If you have any questions about your rights as a participant in a research project, or questions, concerns or complaints about the research and wish to speak with someone who is not a member of the research team, you should contact (anonymously, if you wish) the Institutional Review Board, Office of Research, SMU Administrative Building, 81 Victoria Street, Singapore 188065, email irb@smu.edu.sg, phone: 68281925.

If you are interested in any of the topics examined in this research, the following readings might be of interest to you.

Baumeister, R. F., & Leary, M. R. (1995). The need to belong: Desire for interpersonal attachments as a fundamental human motivation. *Psychological Bulletin, 117,* 497-529.

Derrick, J. L., Gabriel, S., & Hugenberg, K. (2009). Social surrogacy: How favored television programs provide the experience of belonging. *Journal of Experimental Social Psychology, 45,* 352-362.

Food Essay Coding Instructions

(The food essays were coded by two trained research assistants according to the scheme below. Discrepancies in judgment were resolved by conversation until the two coders agreed on a response.)

INSTRUCTIONS: Please complete the following questions for each essay. Answer the questions to the best of your ability. Enter your responses in the accompanying Excel file labeled "Food Essay Coding." Using the scale provided, for each item enter the number that best represents your answer (except for items that require a typed response). Be sure to VERY CAREFULLY enter all of your responses. That is, make sure you enter the number you want in the appropriate column. The column label for the Excel file appears next to each item below. After you have completed the essay coding, send the completed Excel file (with your first name in the file name) to Jordan via email (jdtroisi@buffalo.edu). Please submit the completed file by INSERT DATE.

NOTE: The essays are NOT in numerical order. Be sure to enter the number that appears directly above each essay. It is very important that you enter the correct subject number for each essay.

1. What is the subject number associated with the essay (Sub)?

2. Primary food item named (Food)?

-If they do not explicitly mention the name of the food, leave this item blank.

3. Did they discuss a single food or multiple foods (MultFood)?

1 – Single food
2 – Multiple foods

4. What kind of food is the primary food (FType)?

1 – Meal food
2 – Snack food
3 – Dessert food
4 – Cannot tell

5. Did they seem engaged when writing about the show or "into" what they were writing (Engage)?

1	2	3	4	5
Not at all	Only a little bit	Somewhat	Very	Extremely

6. Do they talk about their experience eating the food (FoodExp)?

-For Example: How they got the food ready, what they did to make it, if they have a ritual for eating it.

1	2	3
Yes	No	I don’t know/can’t tell

7. Did they describe the food as their favorite or one of their favorites (Fav)?

0 – No
1 – Yes

8. Does the participant say the food is a family tradition (FamTrad)?

0 – No
1 – Yes

9. Does the participant say the food is associated with a holiday (Holiday)?

0 – No
1 – Yes

10. ONLY COMPLETE THIS ITEM IF HOLIDAY MENTIONED: What was the holiday (Holiday2)?

11. Associated with some other some special event (Event)?

-For example: a family member, old friend coming into town, a significant trip.
0 – No
1 – Yes

12. ONLY COMPLETE THIS ITEM IF SPECIAL EVENT MENTIONED: What was the special event (Event2)?

13. Does the participant say the food is culturally significant to them (Culture)?

-Note: make sure the food is culturally SIGNIFICANT. Having a particular Asian soup does not necessarily make it culturally significant. However, if it is the traditional Asian soup consumed for a particular event, then it would be significant.
0 – No
1 – Yes

14. Does the participant say the food is one associated with their past (Past)?

-For example: a food made whenever on break from elementary school
0 – No
1 – Yes

15. Does the participant say the food reminds them of home (Home)?

-For example: “we always made this at home,” “every time I eat this it makes me feel like I’m at home”

0 – No
1 – Yes

16. Does the participant say the food is one they had while growing up (Growing)?

0 – No
1 – Yes

17. Is the taste of the food mentioned (Taste)?

0 – No
1 – Yes

18. ONLY COMPLETE THIS ITEM IF TASTE IS MENTIONED: How favorable/good does the taste seem (TasteRate)?

1	2	3	4	5	6
Very bad	Somewhat bad	Acceptable, neither good nor bad	Somewhat good	Very good	They didn’t say

19. Is the smell/aroma of the food mentioned (Smell)?

0 – No
1 – Yes

20. ONLY COMPLETE THIS ITEM IF SMELL IS MENTIONED: How favorable/good does the smell or aroma seem (SmellRate)?

1	2	3	4	5	6
Very bad	Somewhat bad	Acceptable, neither good nor bad	Somewhat good	Very good	They didn’t say

21. Does the participant indicate that the food is sweet (Sweet)?

0 – No
1 – Yes

22. How sweet is the food “objectively?” (according to you) (cSweet)

1	2	3	4	5	6
Not sweet	A little sweet	Somewhat sweet	Sweet	Very sweet	Don’t know

23. Does the participant indicate that the food is salty (Salty)?

0 – No
1 – Yes

24. How salty is the food "objectively?" (cSalty)

1	2	3	4	5	6
Not salty	A little salty	Somewhat salty	Salty	Very salty	Don't know

25. Does the participant indicate that the food is fatty, NOT necessarily fattening (Fatty)?

-For example: fatty meats, oily foods, greasy foods
0 – No
1 – Yes

26. How fatty is the food "objectively?" (cFatty)

1	2	3	4	5	6
Not fatty	A little fatty	Somewhat fatty	Fatty	Very fatty	Don't know

27. Does the participant indicate that the food is filling (Filling)?

-For example: "fills me up," "makes me not want to eat anymore"
0 – No
1 – Yes

28. How filling is the food "objectively?" (cFilling)

1	2	3	4	5	6
Not filling	A little filling	Somewhat filling	Filling	Very filling	Don't know

29. Does the participant indicate that the food is high in carbs (Carbs)?

-For example: breads, pastas, rice
0 – No
1 – Yes

30. How high in carbs is the food "objectively?" (cCarbs)

1	2	3	4	5	6
No carbs	Low in carbs	Moderate in carbs	High in carbs	Very high in carbs	Don't know

31. At what temperature is the food normally served (Temp)?

0 – Cold

1 – Room temperature
2 – Hot

32. Does the participant point out the food is fattening or otherwise unhealthy (Unhealthy)?

0 – No
1 – Yes

33. Does the participant feel regretful for eating the food (Regret)?

0 – No
1 – Yes

34. Does the participant feel guilty for eating the food (Guilt)?

0 – No
1 – Yes

35. Does the participant indicate they ate too much of the food (TooMuch)?

0 – No
1 – Yes

36. What was the overall tone used to describe the food (FoodTone)?

1	2	3	4	5	6
Very negative	Somewhat negative	Acceptable, neither negative nor positive	Somewhat positive	Very positive	They didn't say

37. What was the overall tone of the essay (EssayTone)?

1	2	3	4	5	6
Very negative	Somewhat negative	Acceptable, neither negative nor positive	Somewhat positive	Very positive	They didn't say

38. Is any other person beside the participant mentioned in the essay (OtherP)?

0 – No
1 – Yes

39. Did the participant prepare or get the food themselves (SelfPrep)?

0 – No
1 – Yes

40. Was the food prepared/given by at least one close other (CloseO)?

-For example: family member, romantic partner, friend.
0 – No
1 – Yes

41. ONLY COMPLETE THIS ITEM IF FOOD IS PREPARED/GAVE BY SOMEONE ELSE: How many close others prepared/gave the food (CloseONum)?

1 – At least 1 who is not the participant
2 – At least 2 who are not the participant
3 – At least 3 who are not the participant
4 – At least 3 who are not the participant
…and so on

42. What is/are the relationship(s) between the participant and the close other(s) who prepared or gave the food (CloseRel)?

-Note: List these, separated by commas. For example: mother, uncle, best friend,

43. How many people ate the food with the participant (OtherNum)?

0 – None
1 – At least 1 who is not the participant
2 – At least 2 who are not the participant
3 – At least 3 who are not the participant
4 – At least 3 who are not the participant
…and so on

44. Are mentions of other people positive or negative in tone (OtherTone)?

1	2	3	4	5	6
Very negative	Somewhat negative	Acceptable, neither negative nor positive	Somewhat positive	Very positive	They didn’t say/no others mentioned

[In order to determine if there were differences in the types of foods people identified as comfort foods vs. new foods in essay 2, we computed a variable called “foodcomp”. This variable was the mean of whether comfort foods were more likely to be identified as a favorite food (“Fav”), a family tradition (“FamTrad”), a cultural tradition (“Culture”), something eaten for a holiday (“Holiday”), something eaten for a significant event (“Event”), a part of the participants past (“Past”), or a reminder of home (“Home”).]

Appendix B

Original set of analyses by Troisi & Gabriel (2011):

	Model	Statistics
3-way ANOVA	2 (Secure vs. Insecure) X 2 (Belongingness Threat vs. Control) X 2 (Comfort vs. New food)	Cohen's f^2= .27[1], F(1, 102) = 7.18, p < .01, η^2 = .07)
3-way ACOVA (controlling for mood)	2 (Secure vs. Insecure) X 2 (Belongingness Threat vs. Control) X 2 (Comfort vs. New food)	Cohen's f^2= .23, F(1, 98) = 5.21, p = < .05, η^2 = .05
2-way ANOVA (for Belongingness Control)	2 (Secure vs. Insecure) X 2 (Comfort vs. New food)	Cohen's f^2= .20, F(1, 52) = 2.38, p = .13, η^2 = .04
2-way ANOVA (for Belongingness Threat)	2 (Secure vs. Insecure) X 2 (Comfort vs. New food)	Cohen's f^2= .33, F(1, 50) = 5.38, p = < .05, η^2 = .10
T-test (for Secure)	Comfort food vs. New Food	Cohen's d = .88, t(25) = 2.25, p = < .05
T-test (for Insecure)	Comfort food vs. New Food	Cohen's d = .46, t(25) = -1.17, p = .25

MTurk Replication:

	Model	Statistics
3-way ANOVA	2 (Secure vs. Insecure) X 2 (Belongingness Threat vs. Control) X 2 (Comfort vs. New food)	Cohen's f^2 = .054, F(1, 358) = 1.08, p = .30, η^2 = .003
3-way ACOVA (controlling for mood)	2 (Secure vs. Insecure) X 2 (Belongingness Threat vs. Control) X 2 (Comfort vs. New food)	Cohen's f^2= .040, F(1, 356) = .57, p = .45, η^2 = .002
2-way ANOVA	2 (Secure vs. Insecure) X 2 (Comfort	Cohen's f^2= .045, F(1, 185) = .46, p

[1] We calculated Cohen's f^2 using GPower. This (and subsequent) Cohen's f^2 was not from the original authors. We calculated this based on their reported η2.

(for Belongingness Control)	vs. New food)	= .48, η^2 =.002
2-way ANOVA (for Belongingness Threat)	2 (Secure vs. Insecure) X 2 (Comfort vs. New food)	Cohen's f^2= .18, F(1, 173) = 5.49, p = .02, η^2 = .031
T-test (for Secure)	Comfort food vs. New Food	Cohen's d = .42, t(77) = 1.84, p = .070
T-test (for Insecure)	Comfort food vs. New Food	Cohen's d = .32, t(96) = -1.57, p = .12

Singapore Replication:

	Model	Statistics
3-way ANOVA	2 (Secure vs. Insecure) X 2 (Belongingness Threat vs. Control) X 2 (Comfort vs. New food)	Cohen's f^2 = .11, F(1, 190) = 2.54, p = .11, η^2 = .013
3-way ACOVA (controlling for mood)	2 (Secure vs. Insecure) X 2 (Belongingness Threat vs. Control) X 2 (Comfort vs. New food)	Cohen's f^2= .045, F(1, 188) = .35, p = .55, η^2 = .002
2-way ANOVA (for Belongingness Control)	2 (Secure vs. Insecure) X 2 (Comfort vs. New food)	Cohen's f^2= .02, F(1, 94) = .04, p = .85, η^2 =.0004
2-way ANOVA (for Belongingness Threat)	2 (Secure vs. Insecure) X 2 (Comfort vs. New food)	Cohen's f^2= .27, F(1, 96) = 7.06, p = .009, η^2 = .07
T-test (for Secure; Belongingness Threat)	Comfort food vs. New Food	Cohen's d = .47, t(35) = 1.38, p = .18
T-test	Comfort food vs. New Food	Cohen's d = .69, t(61) = -2.70, p =

(for Insecure; Belongingness Threat)		.009

Netherlands Replication:

	<u>Model</u>	<u>Statistics</u>
3-way ANOVA	2 (Secure vs. Insecure) X 2 (Belongingness Threat vs. Control) X 2 (Comfort vs. New food)	Cohen's f^2 = .006, *F*(1, 167) = .007, *p* = .93, η^2 = .00004
3-way ACOVA (controlling for mood)	2 (Secure vs. Insecure) X 2 (Belongingness Threat vs. Control) X 2 (Comfort vs. New food)	Cohen's f^2= .045, *F*(1, 165) = .38, *p* = .54, η^2 = .002
2-way ANOVA (for Belongingness Control)	2 (Secure vs. Insecure) X 2 (Comfort vs. New food)	Cohen's f^2= .12, *F*(1, 86) = 1.21, *p* = .28, η^2 =.014
2-way ANOVA (for Belongingness Threat)	2 (Secure vs. Insecure) X 2 (Comfort vs. New food)	Cohen's f^2= .14, *F*(1, 81) = 1.65, *p* = .20, η^2 = .02
T-test (for Secure; Belongingness Threat)	Comfort food vs. New Food	Cohen's *d* = .009, *t*(36) = .03, *p* = .98
T-test (for Insecure; Belongingness Threat)	Comfort food vs. New Food	Cohen's *d* = .48, *t*(45) = 1.61, *p* = .12

Appendix C

Netherlands Replication (Only participants who completed study in psychology lab):

	Model	Statistics
3-way ANOVA	2 (Secure vs. Insecure) X 2 (Belongingness Threat vs. Control) X 2 (Comfort vs. New food)	Cohen's f^2 = .03, F(1, 75) = .11, p = .74, η^2 = .001
3-way ACOVA (controlling for mood)	2 (Secure vs. Insecure) X 2 (Belongingness Threat vs. Control) X 2 (Comfort vs. New food)	Cohen's f^2= .14, F(1, 73) = 1.48, p = .23, η^2 = .02
2-way ANOVA (for Belongingness Control)	2 (Secure vs. Insecure) X 2 (Comfort vs. New food)	Cohen's f^2= .10, F(1, 39) = .24, p = .62, η^2 =.01
2-way ANOVA (for Belongingness Threat)	2 (Secure vs. Insecure) X 2 (Comfort vs. New food)	Cohen's f^2= .02, F(1, 36) = .01, p = .92, η^2 = .0003
T-test (for Secure; Belongingness Threat)	Comfort food vs. New Food	Cohen's d = .009, t(20) = .13, p = .90
T-test (for Insecure; Belongingness Threat)	Comfort food vs. New Food	Cohen's d = .48, t(16) = -.04, p = .97

Netherlands Replication (Only participants who completed study alone):

	Model	Statistics
3-way ANOVA	2 (Secure vs. Insecure) X 2 (Belongingness Threat vs. Control) X 2 (Comfort vs. New food)	Cohen's f^2 = .03, F(1, 153) = .12, p = .73, η^2 = .001
3-way ACOVA (controlling for	2 (Secure vs. Insecure) X 2 (Belongingness Threat vs. Control)	Cohen's f^2= .10, F(1, 151) = 1.39, p = .24, η^2 = .009

mood)	X 2 (Comfort vs. New food)	
2-way ANOVA (for Belong Control)	2 (Secure vs. Insecure) X 2 (Comfort vs. New food)	Cohen's f^2= .10, F(1, 77) = .76, p = .39, η^2 =.01
2-way ANOVA (for Belongingness Threat)	2 (Secure vs. Insecure) X 2 (Comfort vs .New food)	Cohen's f^2= .18, F(1, 76) = 2.35, p = .13, η^2 = .03
T-test (for Secure; Belongingness Threat)	Comfort food vs. New Food	Cohen's d = .009, t(85) = 1.24, p = .22
T-test (for Insecure; Belongingness Threat)	Comfort food vs. New Food	Cohen's d = .48, t(42) = 1.65, p = .11

Appendix D

Netherlands Replication (Only participants who completed study in psychology lab):

The contrasts of interest are:

	Expectation	Group A	Group B
Comparison 1<br>(5 vs. 7)<br>(*p* = .93)	Threatened, securely attached participants should experience a reduction in loneliness if they wrote about comfort food, than if they wrote about new food.	5<br>(*M* = 1.54, *SD* = .32)	7<br>(*M* = 1.56, *SD* = .28)
Comparison 2<br>(7 vs. 8)<br>(*p* = .08)	After thinking or writing about comfort food, threatened, securely attached participants should experience a reduction in loneliness but not for those who were insecurely attached.	7<br>(*M* = 1.56, *SD* = .28)	8<br>(*M* = 1.91, *SD* = .33)
Comparison 3<br>(3 vs. 7)<br>(*p* = .06)	After thinking or writing about comfort food, there should be little or no difference in loneliness level in securely attached participants, whether they experienced belongingness threat or not.	3<br>(*M* = 1.91, *SD* = .41)	7<br>(*M* = 1.56, *SD* = .28)
Comparison 4<br>*new*<br>(1 vs. 5)<br>(*p* = .72)	Those asked to experience belongingness threat should report greater loneliness levels, as compared to those who did not experience the threat.	1<br>(*M* = 1.62, *SD* = .28)	5<br>(*M* = 1.54, *SD* = .32)

Netherlands Replication (Only participants who completed study alone):

The contrasts of interest are:

	Expectation	Group A	Group B
Comparison 1<br>(5 vs. 7)<br>(*p* = .82)	Threatened, securely attached participants should experience a reduction in loneliness if they wrote about comfort food, than if they wrote about new food.	5<br>(*M* = 1.65, *SD* = .28)	7<br>(*M* = 1.65, *SD* = .32)
Comparison 2	After thinking or writing about comfort	7	8

(7 vs. 8)<br>($p < .001$)	food, threatened, securely attached participants should experience a reduction in loneliness but not for those who were insecurely attached.	(M = 1.65,<br>SD = .32)	(M = 2.21,<br>SD = .69)
Comparison 3<br>(3 vs. 7)<br>(p = .32)	After thinking or writing about comfort food, there should be little or no difference in loneliness level in securely attached participants, whether they experienced belongingness threat or not.	3<br>(M = 1.77,<br>SD = .36)	7<br>(M = 1.65,<br>SD = .32)
Comparison 4<br>*new*<br>(1 vs. 5)<br>(p = .95)	Those asked to experience belongingness threat should report greater loneliness levels, as compared to those who did not experience the threat.	1<br>(M = 1.66,<br>SD = .27)	5<br>(M = 1.65,<br>SD = .28)

Appendix E

Here, we list the steps and raw values (up to 3 s.f.) used to calculate and evaluate the replications using Simonsohn's (2013) suggested method. Besides those specified with calculation tool used, all calculations were done manually using a calculator.

As mentioned in manuscript, it is possible to understand the original finding as a set of three findings:

Finding 1: Simple effect – Social threat makes people feel lonelier.

Finding 2: 2x2 interaction – But this is less so if people thought of comfort food. (Attenuation of the simple effect)

Finding 3: 2x2x2 interaction – The 2-way interaction is less prominent for people who have insecure attachment styles. (Attenuation of the attenuated effect).

According to Simonsohn (personal communications, April 2014), it is possible to only replicate a subset of findings. However, as each finding builds on each other, starting from the simple effect, Finding 2 will only make sense if Finding 1 occurs. The same goes for Finding 3 with regards to Findings 2 and 1.

Calculating $d_{33\%}$ for Finding 1:

From the original 3-way F test, we obtained the degrees of freedom ($df_1 = 1$, $df_2 = 102$), as well as the average cell size *n* ($110/8 \approx 14$).

With G*Power, we calculated the noncentrality parameter for a generic F test with $df_1 = 1$, $df_2 = 102$, and power = 33%.

$ncp_{33\% (F)} = 2.353$

After converting to t-test version:

$ncp_{33\% (t)} = 1.534$

Because $ncp_{(t)} = d*sqrt(n/2)$,

$d_{33\%(simple)} = .580$

Calculating $d_{33\%}$ for Finding 2:

For a 2x2 interaction, the formula for $ncp_{(t)} = d*sqrt\ (n/4)$ (Simonsohn, 2014). Since it was found that $ncp_{33\% (t)} = 1.534$, this makes $d_{33\%(2\text{-way})} = .820$.

Calculating $d_{33\%}$ for Finding 3:

For a 2x2x2 interaction, the formula for $_{ncp(t)}$ = d*sqrt (n/8) (Simonsohn, 2014). Since it was found that $ncp_{33\%\,(t)}$ = 1.534, this makes $d_{33\%(2\text{-way})}$ = 1.159.

After calculating the $d_{33\%}$ for each finding, it is possible to evaluate each replicated study we conducted.

Mturk replication:

Total N = 366; each cell *n* = 45.75

Finding 1 (Simple effect):

Using SPSS, we ran t-test analysis with the social threat variable (Belongingness threat) as the grouping variable, and loneliness (Slone) as the dependent variable.

t(364) = -1.406, *p* = .160

Condition	Mean	SD
Belongingness Threat	2.226	.971
Control	2.366	.933

Utilizing DeFife's (2009) excel calculator, d_{rep} = .15.

Since $d_{33\%(simple)}$ = .580, $ncp_{33\%}$[2] = √[(45.75)/2] * .58 = 2.774

Using R to evaluate the probability of observing a t-statistic that is ≤ -1.406,

pt (-1.406, df = 364, ncp = 2.774) → *p* = .001.

This indicates that we reject the null of a detectable ($d_{33\%}$) effect; implying that the effect we were trying to detect was smaller than small.

Finding 2 (2x2 interaction effect):

Using SPSS, we ran F-test analysis for 2 (Belongingness Threat vs Control) X 2 (Comfort food vs New food) with loneliness (Slone) as the dependent variable.

t(362) = .440, *p* = .66

Condition	Mean	SD
Comfort Food		

[2] $ncp_{33\%}$ indicates the ncp for null of $d_{33\%}$, for the sample size of the replication.

	Control	2.195	.976
	Belongingness Threat	2.366	.968
New Food			
	Control	2.287	.965
	Belongingness Threat	2.365	.866

Total variance = 3.571; Pooled *SD* = .472

Since $d_{33\%(2\text{-}way)}$ = .820, $ncp_{33\%} = \sqrt{[(45.75)/4]} * .82 = 2.773$

Replicated $d_{rep} = d_1 - d_2 = .197$

$d_1 = (2.366 - 2.195) / (.472) = .362$

$d_2 = (2.365 - 2.287) / (.472) = .165$

Using R to evaluate the probability of observing a t-statistic that is ≤ .440,

pt (.440, df = 362, ncp = 2.773) → *p* = .010.

This indicates that we reject the null of a detectable ($d_{33\%}$) effect; implying that the effect we were trying to detect was smaller than small.

Finding 3 (2x2x2 interaction effect):

Using SPSS, we ran F-test analysis for 2 (Belongingness Threat vs Control) X 2 (Comfort food vs New food) X 2 (Secure vs Insecure attachment style) with loneliness (Slone) as the dependent variable.

$t(358) = 1.037$, $p = .301$

Condition			Mean	SD
Secure				
	Comfort Food			
		Control	1.571	.576
		Belongingness Threat	1.811	.716
	New Food			
		Control	1.697	.669
		Belongingness Threat	2.138	.773
Insecure				
	Comfort Food			

	Control	2.554	.980
	Belongingness Threat	2.834	.907
New Food			
	Control	2.472	.975
	Belongingness Threat	2.532	.903

Total variance = 5.439; Pooled *SD* = .292

Since $d_{33\%(2\text{-}way)}$ = 1.159, $ncp_{33\%}$ = √[(45.75)/8] * 1.159 = 2.772

Replicated $d_{rep} = (d_1 - d_2) - (d_3 - d_4)$ = -1.445

d_1 = (1.811 – 1.571) / (.292) = .825

d_2 = (2.138 – 1.697) / (.292) = 1.514

d_3 = (2.834 – 2.554) / (.292) = .963

d_4 = (2.532 – 2.472) / (.292) = .207

Using R to evaluate the probability of observing a t-statistic that is ≤ 1.037,
pt (1.037, df = 358, ncp = 2.772) → *p* = .041.
This indicates that we reject the null of a detectable ($d_{33\%}$) effect; implying that the effect we were trying to detect was smaller than small.

Singapore replication:

Total N = 198; each cell *n* = 24.75

Finding 1 (Simple effect):

Using SPSS, we ran t-test analysis with the social threat variable (Belongingness threat) as the grouping variable, and loneliness (Slone) as the dependent variable.

$t(196) = .516$, $p = .606$

Condition	Mean	SD
Belongingness Threat	2.359	.762
Control	2.302	.790

Utilizing DeFife's (2009) excel calculator, d_{rep} = .074.

Since $d_{33\%(simple)}$ = .580, $ncp_{33\%}$ = √[(24.75)/2] * .58 = 2.040

Using R to evaluate the probability of observing a t-statistic that is ≤ .516,

pt (.516, df = 196, ncp = 2.040) → *p* = .064.

This indicates that we do not reject the null of a detectable ($d_{33\%}$) effect, although this was marginal. This means that our replication do not tell whether the effect we were trying to test was smaller than small.

Finding 2 (2x2 interaction effect):

Using SPSS, we ran F-test analysis for 2 (Belongingness Threat vs Control) X 2 (Comfort food vs New food) with loneliness (Slone) as the dependent variable.

$t(194) = 1.435$, $p = .153$

Condition		Mean	SD
Comfort Food			
	Control	2.339	.750
	Belongingness Threat	2.239	.646
New Food			
	Control	2.267	.832
	Belongingness Threat	2.484	.855

Total variance = 2.404; Pooled *SD* = .388

Since $d_{33\%(2\text{-}way)} = .820$, $ncp_{33\%} = \sqrt{[(24.75)/4]} * .82 = 2.040$

Replicated $d_{rep} = d_1 - d_2 = -.817$

$d_1 = (2.239 - 2.339) / (.388) = -.258$

$d_2 = (2.484 - 2.267) / (.388) = .559$

Using R to evaluate the probability of observing a t-statistic that is ≤ 1.435,

pt (1.435, df = 194, ncp = 2.039) → *p* = .273.

This indicates that we do not reject the null of a detectable ($d_{33\%}$) effect.

Finding 3 (2x2x2 interaction effect):

Using SPSS, we ran F-test analysis for 2 (Belongingness Threat vs Control) X 2 (Comfort food vs New food) X 2 (Secure vs Insecure attachment style) with loneliness (Slone) as the dependent variable.

$t(190) = 1.593$, $p = .113$

Condition	Mean	SD

Secure				
	Comfort Food			
		Control	2.020	.489
		Belongingness	2.108	.575
		Threat		
	New Food			
		Control	1.921	.590
		Belongingness	1.880	.435
		Threat		
Insecure				
	Comfort Food			
		Control	2.483	.808
		Belongingness	2.317	.682
		Threat		
	New Food			
		Control	2.446	.889
		Belongingness	2.837	.843
		Threat		

Total variance = 3.723; Pooled *SD* = .241

Since $d_{33\%(2\text{-}way)}$ = 1.159, $ncp_{33\%}$ = √[(45.75)/8] * .1.159 = 2.772

Replicated $d_{rep} = (d_1 - d_2) - (d_3 - d_4)$ = 2.867

d_1 = (2.108 – 2.02) / (.241) = .365

d_2 = (1.875 – 1.921) / (.241) = -.191

d_3 = (2.317 – 2.483) / (.241) = -.689

d_4 = (2.837 – 2.446) / (.241) = 1.622

Using R to evaluate the probability of observing a t-statistic that is ≤ 1.593,
pt (1.593, df = 190, ncp = 2.867) → *p* = .102.
This indicates that we do not reject the null of a detectable ($d_{33\%}$) effect.

Netherlands replication:

Total N = 175; each cell *n* = 21.87

Finding 1 (Simple effect):

Using SPSS, we ran t-test analysis with the social threat variable (Belongingness threat) as the grouping variable, and loneliness (Slone) as the dependent variable.

$t(173) = -1.106$, $p = .270$

Condition	Mean	SD
Belongingness Threat	1.858	.527
Control	1.951	.575

Utilizing DeFife's (2009) excel calculator, $d_{rep} = .168$.

Since $d_{33\%(simple)} = .580$, $ncp_{33\%} = \sqrt{[(21.87)/2]} * .58 = 1.918$

Using R to evaluate the probability of observing a t-statistic that is ≤ -1.106,

pt (-1.106, df = 173, ncp = 1.918) → $p = .001$.

This indicates that we reject the null of a detectable ($d_{33\%}$) effect; implying that the effect we were trying to detect was smaller than small.

Finding 2 (2x2 interaction effect):

Using SPSS, we ran F-test analysis for 2 (Belongingness Threat vs Control) X 2 (Comfort food vs New food) with loneliness (Slone) as the dependent variable.

$t(171) = .145$, $p = .865$

Condition		Mean	SD
Comfort Food			
	Control	2.000	.662
	Belongingness Threat	1.925	.611
New Food			
	Control	1.897	.463
	Belongingness Threat	1.793	.426

Total variance = 1.207; Pooled $SD = .275$

Since $d_{33\%(2\text{-}way)} = .820$, $ncp_{33\%} = \sqrt{[(21.87)/4]} * .82 = 1.917$

Replicated $d_{rep} = d_1 – d_2 = .106$

$d_1 = (1.925 – 2.000) / (.275) = -.272$

$d_2 = (1.793 – 1.897) / (.275) = -.378$

Using R to evaluate the probability of observing a t-statistic that is ≤ .145,

pt (.145, df = 171, ncp = 1.917) → *p* = .273.

This indicates that we do not reject the null of a detectable ($d_{33\%}$) effect.

Finding 3 (2x2x2 interaction effect):

Using SPSS, we ran F-test analysis for 2 (Belongingness Threat vs Control) X 2 (Comfort food vs New food) X 2 (Secure vs Insecure attachment style) with loneliness (Slone) as the dependent variable.

$t(167) = .155$, $p = .934$

Condition			Mean	SD
Secure				
	Comfort Food			
		Control	1.742	.362
		Belongingness Threat	1.650	.355
	New Food			
		Control	1.706	.294
		Belongingness Threat	1.647	.279
Insecure				
	Comfort Food			
		Control	2.319	.806
		Belongingness Threat	2.175	.691
	New Food			
		Control	2.034	.518
		Belongingness Threat	1.898	.485

Total variance = 2.051; Pooled *SD* = .179

Since $d_{33\%(2\text{-}way)} = 1.159$, $ncp_{33\%} = \sqrt{[(21.87)/8]} * 1.159 = 1.916$

Replicated $d_{rep} = (d_1 - d_2) - (d_3 - d_4) = -.140$

$d_1 = (1.650 - 1.742) / (.179) = -.514$

$d_2 = (1.647 - 1.706) / (.179) = -.330$

$d_3 = (2.175 - 2.319) / (.179) = -.804$

$d_4 = (1.898 - 2.034) / (.179) = -.760$

Using R to evaluate the probability of observing a t-statistic that is ≤ .155,

pt (.155, df = 167, ncp = 1.916) → *p* = .039.

This indicates that we reject the null of a detectable ($d_{33\%}$) effect.
